# Editorial

**Published:** 2016

**Authors:** Zemba Mihail

**Affiliations:** * Editor in Chief, Romanian Journal of Ophthalmology

Romanian Journal of Ophthalmology has already reached the age of one year and it is my duty as the Editor in Chief to assess the achievements and also the non-achievements of our review.

The aim of changing Oftalmologia in Romanian Journal of Ophthalmology, in other words switching from publishing in Romanian to publishing in English, was to obtain a better visibility and quotation.

The “new look” of the Romanian Journal of Ophthalmology was greatly appreciated by everyone. Regarding the quality of the graphic design, as well as that of the paper and of the pictures, this has improved; especially in that we succeeded in publishing the entire iconography in color.

The Editorial Board established a standard format of article editing, which was respected by the authors. We will keep on mentioning these details (i.e. complete name and surname of the authors, address of the corresponding author, respecting the IMRAD form: introduction, methods, results and discussion, including acknowledgements, disclosures and references), because these elements are important for the quotation process.

We succeeded in publishing four issues of our review between May and December 2015, a necessary condition for quotation. It was a great effort for the ophthalmologic community, but this effort must continue; it is mandatory to publish an issue at every three months.

A web site for our journal is already available: www.rjo.ro. The electronic version of our review can be found by anyone when accessing the link, with a delay of one issue behind the printed version, together with all the information needed for publishing an article, under the title “Guidelines for authors”. This is another method to increase the international visibility of our review and also a necessary condition for a favorable quotation.

Initially, there were some concerns about publishing in English and I am sure that this was not the ideal solution, but as time passes results start to show and I can only hope they continue. It was very difficult to publish Oftalmologia due to the lack of articles and the Board of Oftalmologia and of the Romanian Society of Ophthalmology had to make a difficult decision: either to continue publishing in Romanian, which would have resulted into having no review, so, the end of it, or make a change. With the risk of being redundant, I wish to reinforce the fact that in the past year we have managed to publish an issue at every two months.

The effort of indexing our review in some of the most important international databases carries on. It is a process more difficult than we expected. In addition, for the assessment of our journal in the databases we were required to send the last four issues (or the last 50 articles), these being sent at the beginning of this year, and the evaluation process takes some months.

We look forward to announcing the indexing of Romanian Journal of Ophthalmology in some important international databases (such as PubMed) in one of the future issues.

**Mihail Zemba** **Editor in Chief**

